# Outcome prediction of SSTR-RADS-3A and SSTR-RADS-3B lesions in patients with neuroendocrine tumors based on ^68^Ga-DOTATATE PET/MR

**DOI:** 10.1007/s00432-024-05776-5

**Published:** 2024-05-25

**Authors:** Jing Gao, Jinxin Zhou, Chang Liu, Yu Pan, Xiaozhu Lin, Yifan Zhang

**Affiliations:** Department of Nuclear Medicine, Ruijin Hospital, Shanghai Jiao Tong University School of Medicine, No. 197, Ruijin 2nd Road, Shanghai, 200025 China

**Keywords:** Neuroendocrine tumors, PET/MR, ^68^Ga-DOTATATE, SSTR-RADS-3A, SSTR-RADS-3B

## Abstract

**Purpose:**

Somatostatin receptor (SSTR)-targeted PET imaging has emerged as a common approach to evaluating those patients with well-differentiated neuroendocrine tumors (NETs). The SSTR reporting and data system (SSTR-RADS) version 1.0 provides a means of categorizing lesions from 1 to 5 according to the likelihood of NET involvement, with SSTR-RADS-3A (soft-tissue) and SSTR-RADS-3B (bone) lesions being those suggestive of but without definitive NET involvement. The goal of the present study was to assess the ability of ^68^Ga-DOTATATE PET/MR imaging data to predict outcomes for indeterminate SSTR-RADS-3A and 3B lesions.

**Methods:**

NET patients with indeterminate SSTR-RADS-3A or SSTR-RADS-3B lesions who underwent ^68^Ga-DOTATATE PET/MR imaging from April 2020 through August 2023 were retrospectively evaluated. All patients underwent follow-up through December 2023 (median, 17 months; (3–31 months)), with imaging follow-up or biopsy findings ultimately being used to classify lesions as malignant or benign. Lesion maximum standardized uptake value (SUVmax) along with minimum and mean apparent diffusion coefficient (ADCmin and ADCmean) values were measured and assessed for correlations with outcomes on follow-up.

**Results:**

In total, 33 indeterminate SSTR-RADS-3 lesions from 22 patients (19 SSTR-RADS-3A and 14 SSTR-RADS-3B) were identified based upon baseline ^68^Ga-DOTATATE PET/MR findings. Over the course of follow-up, 16 of these lesions (48.5%) were found to exhibit true NET positivity, including 9 SSTR-RADS-3A and 7 SSTR-RADS-3B lesions. For SSTR-RADS-3A lymph nodes, a diameter larger than 0.7 cm and an ADCmin of 779 × 10^−6^mm^2^/s or lower were identified as being more likely to be associated with metastatic lesions. Significant differences in ADCmin and ADCmean were identified when comparing metastatic and non-metastatic SSTR-RADS-3B bone lesions (P < 0.05), with these parameters offering a high predictive ability (AUC = 0.94, AUC = 0.86).

**Conclusion:**

Both diameter and ADCmin can aid in the accurate identification of the nature of lesions associated with SSTR-RADS-3A lymph nodes, whereas ADCmin and ADCmean values can inform the accurate interpretation of SSTR-RADS-3B bone lesions.

**Supplementary Information:**

The online version contains supplementary material available at 10.1007/s00432-024-05776-5.

## Introduction

Neuroendocrine tumors (NETs) are a heterogeneous tumor type that can arise from anatomical locations with diverse biological behaviors (Kulke et al. 2011). Well-differentiated NETs exhibit characteristic cell membrane somatostatin receptor (SSTR) expression, providing a basis for the development of radiolabeled SSTR analogs with affinity for these cells (Geijer and Breimer 2013), thereby enabling functional DOTATATE PET imaging of these NET lesions. Lymph node (LN) metastasis or distant metastasis is present in roughly half of all NET patients on initial diagnosis (Dasari et al. 2017), and the ability to accurately identify metastatic lesions is vital for effective clinical management, including the selection of appropriate local and systemic treatment options (Kim et al. 2010).

SSTR-RADS imaging strategies have offered promise as an approach to more accurately interpreting lesions exhibiting DOTATATE uptake, facilitating the staging of NET patients, and associated treatment planning (Werner et al. 2021; Weich et al. 2022; Grawe et al. 2023). Those lesions classified in the SSTR-RADS-3 category exhibit indeterminate characteristics that often require additional work-up, which can include follow-up imaging or biopsy (Werner et al. 2018). SSTR-RADS-3 lesions exhibit levels of uptake roughly equivalent to that of the blood pool and lack any distinct anatomical abnormalities, complicating efforts to determine whether lymph node (3A) or bone (3B) metastases are present based solely on uptake data. If these lesions are interpreted as being metastatic without sufficient evidence, this can subject patients to unneeded interventions. Moreover, biopsy is an invasive method and is significantly dependent on the location and depth of the lesion. Therefore, accurate, timely, and non-invasive diagnostic imaging is crucial to ascertain the benign or malignant nature of the lesion before proceeding with treatment.

There is potential value in exploring the performance of different magnetic resonance (MR) parameters as tools for differentiating between benign and malignant lesions in patients with indeterminate ^68^Ga-DOTATATE PET findings. PET/MR imaging combines the unique benefits of both PET and MR imaging strategies to offer both structural and functional insights. MRI imaging-based diffusion-weighted imaging (DWI) is a parameter that serves as a surrogate for the density of tumor cells and can be quantified using the apparent diffusion coefficient (ADC) (Schmid-Tannwald et al. 2013). As tumors generally exhibit higher cell density and more abundant intercellular and intracellular membranes, they generally exhibit ADC values that are lower than those of healthy tissues (Choi et al. 2007). Minimum ADC (ADCmin) values have been leveraged to aid in the detection of metastatic LN involvement (Kim et al. 2008) and bone metastatic lesions (Subhawong et al. 2014). ADC thus offers promising utility as an approach to achieving superior diagnostic accuracy when evaluating indeterminate SSTR-RADS-3A and 3B lesions.

This study was developed to assess the predictive performance of ^68^Ga-DOTATATE PET/MR-derived size and ADC parameters when assessing SSTR-RADS-3A and 3B lesions to determine the presence of underlying malignancy.

## Materials

### Patients

This was a retrospective analysis that enrolled consecutive patients with a confirmed history of pathologically diagnosed NETs who had undergone ^68^Ga-DOTATATE PET/MR scans from April 2020 through August 2023. Our institutional ethics committee approved this study, waiving the need for written informed consent due to the retrospective nature of the study. Patient clinical and demographic data including age, gender, imaging purpose, and location of metastasis were obtained for analysis.

### PET/MR imaging

Patients were intravenously administered 2 MBq/kg of ^68^Ga-DOTATATE, and scanning was performed 45 to 60 min post-injection using a Biograph mMR combined 3.0 T PET/MRI system (Siemens Healthineers, Erlangen, Germany). Total body scanning from the base of the skull to the upper thighs was performed with patients in the supine position. PET image reconstruction was performed using a Gaussian smoothing kernel with an FWHM of 2 mm and a three-dimensional attenuation-weighted ordered-subset expectation maximization (2 iterations, 21 subsets, 172 × 172 matrix) algorithm. MR imaging was performed at the same time as PET scanning and consisted of a T1WI-Dixon sequence, an axial T2-weighted half-Fourier acquisition single-shot turbo spin-echo sequence, and an axial echo-planar DWI sequence (b-value = 50, 800 s/mm^2^). A single exponential function was employed to calculate ADC values.

### Imaging analysis

Two experienced readers (X.Z.L and Y.F.Z) who remained blinded to final patient diagnostic outcomes were responsible for reviewing all ^68^Ga-DOTATATE PET/MR scans, with lesions being categorized as per the SSTR-RADS version 1.0 criteria (Werner et al. 2018). Reviewers reached a unanimous consensus for all lesions included in this study. SSTR-RADS-3A lesions correspond to soft-tissue or LN findings with equivocal uptake (comparable to or slightly higher than the signal for the blood pool), while SSTR-RADS-3B lesions correspond to areas of bone exhibiting low-level uptake.

LIFEx v5.1 (Nioche et al. 2018) was used to measure LN size and other imaging parameters. LN size was measured based on the maximum short-axis diameter on T2w PET/MR images. ADCmean and ADCmin values for SSTR-RADS 3A and 3B lesions were computed by using DWI images to draw a region of interest surrounding the target lesion and transferring this to the ADC map. SUVmax values for SSTR-RADS-3A and 3B lesions were calculated based on a volume of interest (VOI) manually placed around the lesion area containing the pixel with the highest SUV.

### Reference standard

Longitudinal follow-up imaging data from the patients in this study with SSTR-RADS-3A or SSTR-RADS-3B lesions were evaluated, with only those patients who underwent follow-up imaging a minimum of 3 months following baseline ^68^Ga-DOTATATE PET/MR being retained for further analysis. Follow-up imaging strategies employed for these lesions included ^68^Ga-DOTATATE PET/MRI, diagnostic computed tomography (CT), or MR imaging. Lesions identified over the course of follow-up were regarded as being suggestive of NETs if they met a minimum of one of the following criteria:

1. For SSTR-RADS-3A and SSTR-RADS-3B lesions, longitudinal follow-up ^68^Ga-DOTATATE PET/MR imaging results showing increased or decreased radiotracer uptake with a change in SUVmax exceeding 30% (similar to the PERCIST criteria (Garcia-Carbonero et al. 2015)).
2. For SSTR-RADS-3A lesions, the presence of LN metastasis was assessed based on LN size, LN morphology, and the signal characteristics identified through follow-up CT or MR imaging (Loch et al. 2020).
3. For SSTR-RADS-3B lesions, follow-up CT results were assessed for new areas of osteolytic changes or sclerosis, or for more extensive sclerosis in regions previously exhibiting limited or equivocal sclerotic changes. Increases in the size of these lesions or changes in their characteristics from lytic to sclerotic were considered indicative of metastasis (Xie et al. 2022).

### Statistical analysis

R (v 4.2.2) was used for all statistical analyses. Descriptive statistics were employed to summarize patient demographics and baseline clinical characteristics. Categorical and continuous variables were presented as percentages and medians with interquartile ranges, respectively. Mann–Whitney tests were used to compare continuous variables. Receiver operating characteristic (ROC) curves were employed to assess sensitivity and specificity, area under the curve (AUC) values, and the optimal threshold for use when distinguishing between lesions that were benign and malignant. P < 0.05 was regarded as the significance threshold.

## Results

### Patients

Of 168 patients evaluated for potential study inclusion, 36 patients (21.4%) had at least one SSTR-RADS-3A or SSTR-RADS-3B lesion identified on initial ^68^Ga-DOTATATE PET/MR. Of these 36 cases, however, sufficient radiological follow-up was lacking in 14 cases (38.9%), precluding any longitudinal assessment of the target lesions from these patients. Of the 22 patients for whom follow-up results were available, 14 (63.6%) underwent restaging and 8 (36.4%) underwent staging scans. Primary lesions included pancreatic NETs in 17 cases, rectal NETs in 3 cases, liver NETs in 1 case, and lung NETs in 1 case. Over the course of follow-up, 10 of these 22 patients underwent chest/abdominal/pelvic CT scans, 4 underwent ^68^Ga-DOTATATE PET/MR scans, 3 underwent pelvic MR imaging, 3 underwent biopsy procedures, 1 patient underwent a ^68^Ga-DOTATATE PET/CT scan, and 1 underwent neck ultrasound. The median follow-up interval for these patients was 17 months (range: 3–31 months). For details regarding the demographic and clinical characteristics of these patients, see Table 1.

**Table 1:** Patient characteristics

patient	Age (y)	Sex	Aim of imaging	Location of primary lesion	Follow-up	Location of Metastasis
1	67	Female	Restaging	Pancreas	CT	Liver
2	31	Female	Restaging	Pancreas	Biopsy	Bone
3	29	Female	Staging	Pancreas	Biopsy	None
4	37	Female	Restaging	Pancreas	MR	Bone
5	60	Male	Restaging	Pancreas	PET/MR	None
6	45	Female	Restaging	Rectal	MR	None
7	46	Female	Restaging	Lung	PET/MR	Bone
8	36	Female	Restaging	Pancreas	Ultrasound	None
9	55	Female	Staging	Pancreas	PET/MR	None
10	65	Male	Restaging	Pancreas	CT	None
11	48	Male	Staging	Rectal	CT	None
12	59	Male	Restaging	Pancreas	CT	None
13	55	Female	Restaging	Rectal	Biopsy	None
14	67	Male	Restaging	Pancreas	PET/MR	Liver
15	52	Male	Staging	Pancreas	CT	None
16	48	Male	Staging	Pancreas	CT	None
17	72	Male	Restaging	Liver	CT	Liver, bone
18	50	Female	Staging	Pancreas	MR	Liver
19	48	Male	Restaging	Pancreas	CT	Liver, bone
20	59	Male	Staging	Pancreas	CT	None
21	35	Male	Restaging	Pancreas	PET/CT	Bone
22	53	Female	Staging	Pancreas	CT	Bone

### SSTR-RADS-3A and 3B lesions incidence and interpretation

The 22 patients with available longitudinal follow-up data presented with 33 total SSTR-RADS-3A or SSTR-RADS-3B lesions. These included 19 SSTR-RADS-3A LNs (8 retroperitoneal LNs, 4 pelvic LNs, 4 supraclavicular LNs, 3 mediastinal LNs) and 14 SSTR-RADS-3B lesions (7 vertebral lesions, 3 rib lesions, 3 iliac lesions, and 1 hip bone lesion).

**Fig. 1:**
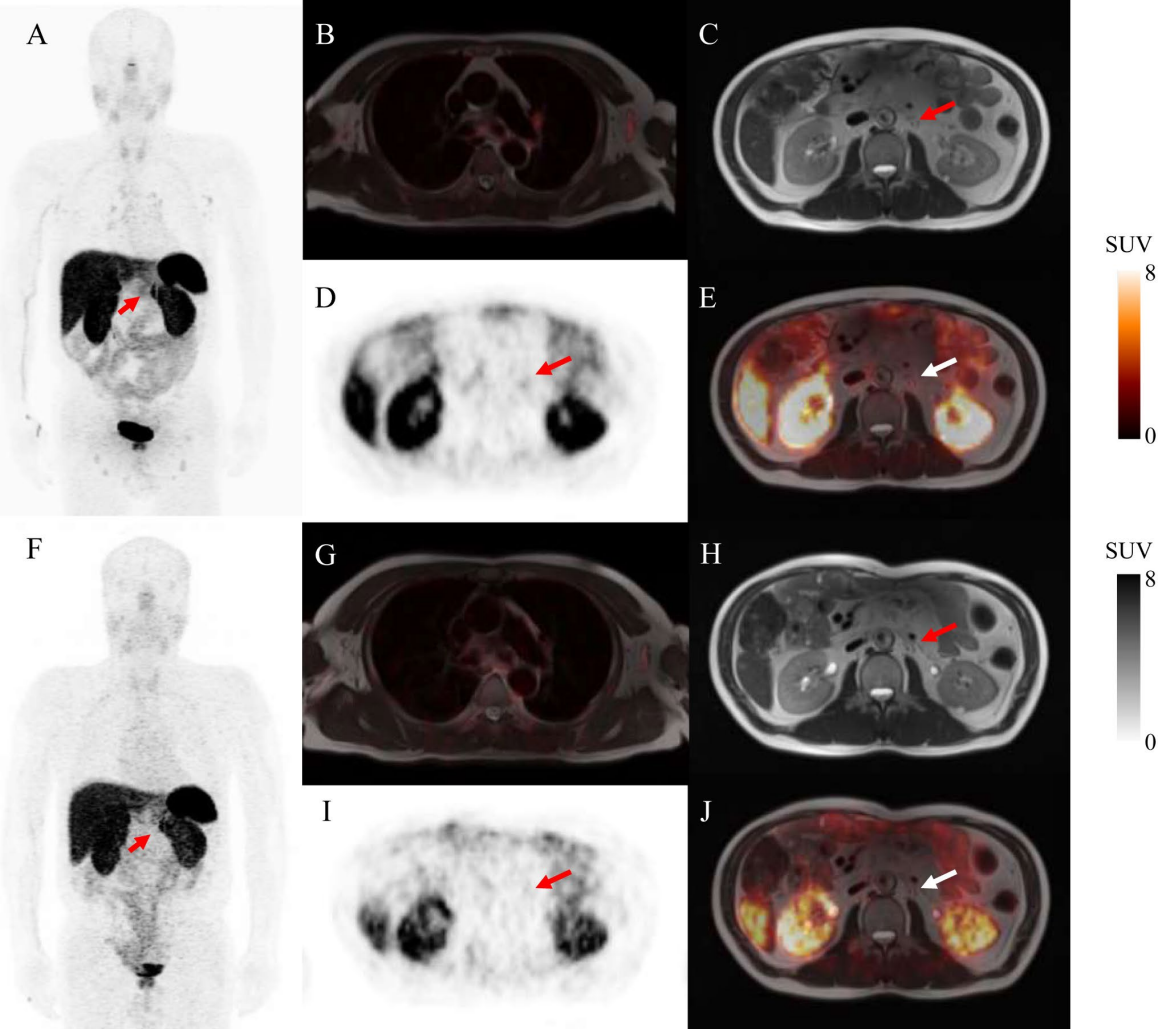
Equivocal SSTR-targeted PET/MR uptake in soft-tissue lesions. ^68^Ga-DOTATATE PET/MR imaging was performed for the restaging of a 60-year-old male with a history of pancreatic NET. Whole-body maximum intensity projection images (MIP) revealed multiple LN metastases with radiotracer uptake that was either pathologic or equivocal (**A**). PET/MR-fused images showing blood pool uptake with a SUVmax of 1.05 (**B**). Axial T2 weighted MR image (**C**), PET image (**D**), and fused PET/MR image (**E**) results revealed a retroperitoneal LN with only slightly higher radiotracer uptake than the blood pool (arrows, SUVmax: 2.9). Follow-up MIP (**F**), blood pool uptake (**G**), axial T2 weighted MR image (**H**), axial ^68^Ga-DOTATATE PET (**I**), and axial ^68^Ga-DOTATATE PET/MR (**J**) scans performed 28 months after initial imaging and treatment showed no visible radiotracer uptake on the follow-up scan (arrows), consistent with likely NET involvement

**Fig. 2:**
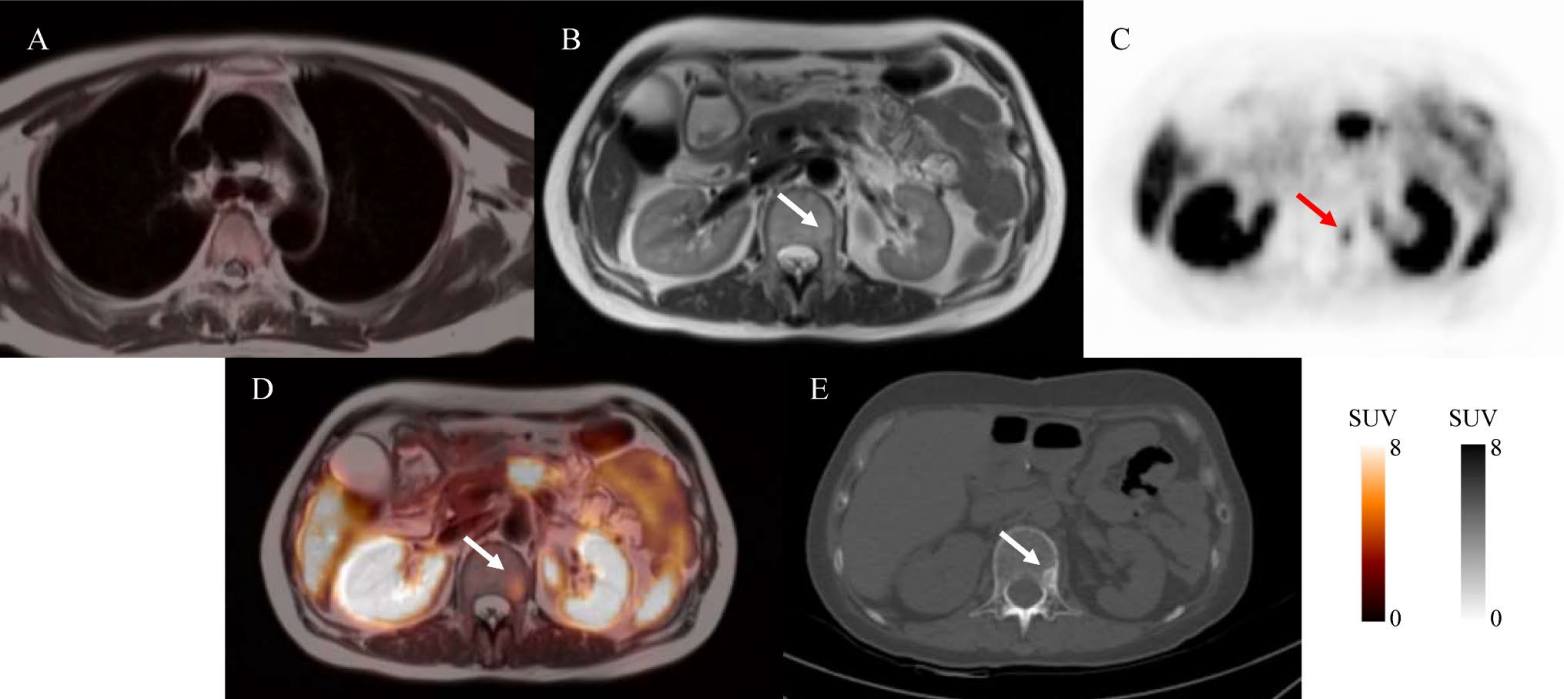
PET/MR-fused images showing blood pool uptake a SUVmax of 0.7 (**A**). Axial T2 weighted MR (**B**), axial ^68^Ga-DOTATATE PET (**C**), and axial ^68^Ga-DOTATATE PET/MR (**D**) images through the pelvis revealing a hypointense mass, DOTATATE-avid uptake in the L1 vertebrae (arrows, SUVmax:5.8). Follow-up CT image performed 22 months after initial imaging (**E**) revealed a pronounced new osteogenic L1 vertebral lesion (arrows) consistent with NET bone metastasis

When outcomes were evaluated at the patient level, 10 of these 22 patients (45.5%) presented with at least 1 SSTR-RADS-3A or SSTR-RADS-3B lesion with imaging changes consistent with malignancy throughout follow-up. Overall, 16 of the 22 patients (72.7%) presented with a single SSTR-RADS-3A or SSTR-RADS-3B lesion, while 6 (27.3%) presented with 2 or more lesions, including 2 patients with both SSTR-RADS-3A and SSTR-RADS-3B lesions. Of the evaluated SSTR-RADS-3A lesions, 9/19 (47.4%) exhibited imaging changes at follow-up consistent with true-positive NET involvement (Fig. 1). The median SUVmax values for the metastatic and non-metastatic lesions were 3.5 (range: 2.6–4.3) and 3.5 (range: 2.5–4.8), respectively (P = 0.9). Of the evaluated SSTR-RADS-3B lesions, 7/14 lesions (50.0%) exhibited follow-up imaging changes consistent with true-positive NET involvement (Fig. 2). The median SUVmax values for the metastatic and non-metastatic lesions were 4.8 (range: 3.1–6.7) and 3.4 (range: 2.7–4.4), respectively (P = 0.54). For further details regarding lesion uptake characteristics, see Table 2.

**Table 2:** Characteristics of SSTR-RADS-3A and SSTR-RADS-3B Lesions

	3A		P value	3B		P value
	Metastatic	Non-metastatic		Metastatic	Non-metastatic	
No. of lesions	9	10		7	7	
SUVmax	3.5 (2.6–4.3)	3.5 (2.5–4.8)	0.90	4.8 (3.1–6.7)	3.4 (2.7–4.4)	0.54
ADCmin (10^−6^mm^2^/s)	584 ± 110	974 ± 192	** < 0.001**	618 ± 204	1077 ± 325	**0.004**
ADCmean(10^−6^mm^2^/s)	1200 ± 244	1393 ± 349	0.28	954 ± 340	1442 ± 396	**0.03**

SUVmax values are presented as median (range) and ADC values are presented as mean ± SD

### Lesion imaging features predictive of malignancy

**Fig. 3:**
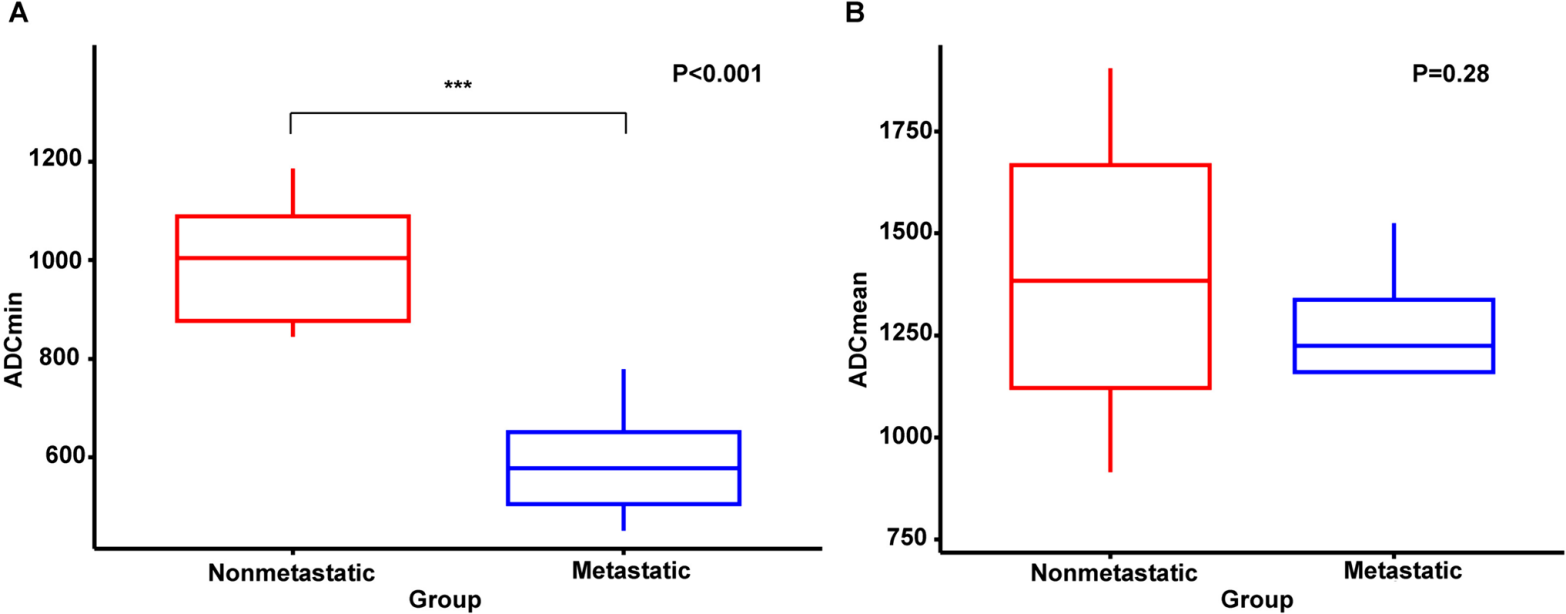
Boxplots corresponding to **A** ADCmin and **B** ADCmean values for metastatic and non-metastatic LNs. The middle line corresponds to the median, while the bars denote the 5th and 95th percentiles, respectively. *ADC* apparent diffusion coefficient, *LN* lymph node

Both LN size and LN ADC values were ultimately found to be capable of predicting whether SSTR-RADS-3A lesions were benign or malignant in this patient cohort. Specifically, the median LN short-axis diameters in the metastatic and non-metastatic groups were 1.0 cm (range: 0.8–2.0 cm) and 0.65 cm (range: 0.5–1.5 cm), respectively (P < 0.05). ROC curves were implemented to determine the optimal LN size for use when differentiating between metastatic and non-metastatic LNs, revealing an optimal threshold of 0.7 cm. Using this threshold, the calculated AUC was 0.78, with respective sensitivity and specificity values of 100.0% and 60.0%, respectively (P = 0.01) (Supplemental Fig. 1). Metastatic LNs exhibited lower ADCmin and ADCmean values as compared to the non-metastatic group (Fig. 3). A highly significant difference in ADCmin values was observed when comparing metastatic and non-metastatic LNs (584 ± 110 × 10^−6^ mm^2^/s vs. 974 ± 192 × 10^−6^mm^2^/s, respectively, P < 0.001). The AUC when using this parameter to distinguish between metastatic and nonmetastatic LNs was 0.93 (Supplemental Fig. 2). ROC curves led to the selection of an ADCmin of 779 × 10^−6^mm^2^/s as the optimal cut-off to distinguish between metastatic and non-metastatic LNs. There was no significant difference in ADCmean values when comparing metastatic and non-metastatic LNs (1200 ± 244 × 10^−6^mm^2^/s and 1393 ± 349 × 10^−6^mm^2^/s, respectively, P = 0.28).

**Fig. 4:**
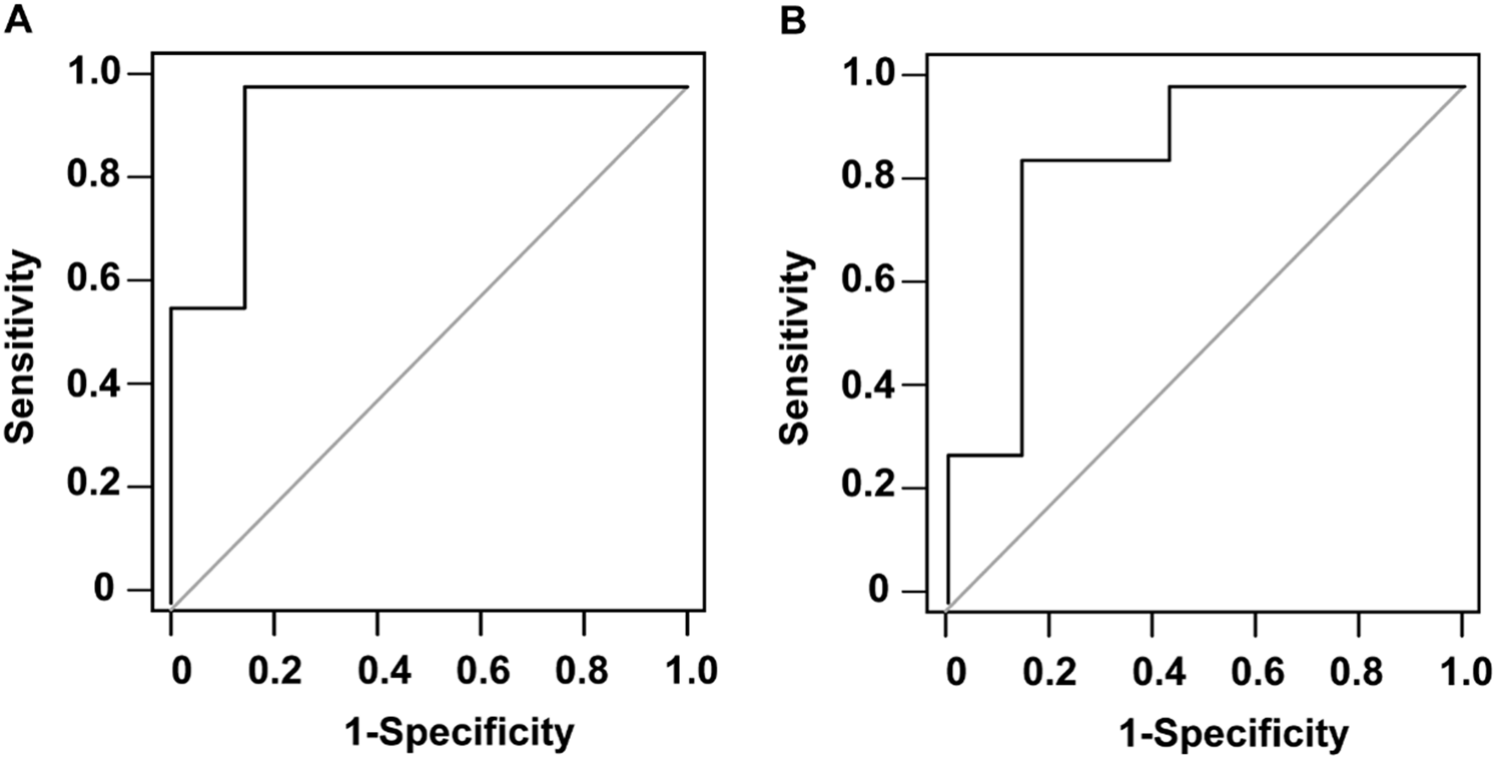
The diagnostic performance of different parameters when differentiating between metastatic and non-metastatic bone lesions. **A** ROC curve for ADCmin (AUC = 0.94). **B** ROC curve for ADCmean (AUC = 0.86)

**Fig. 5:**
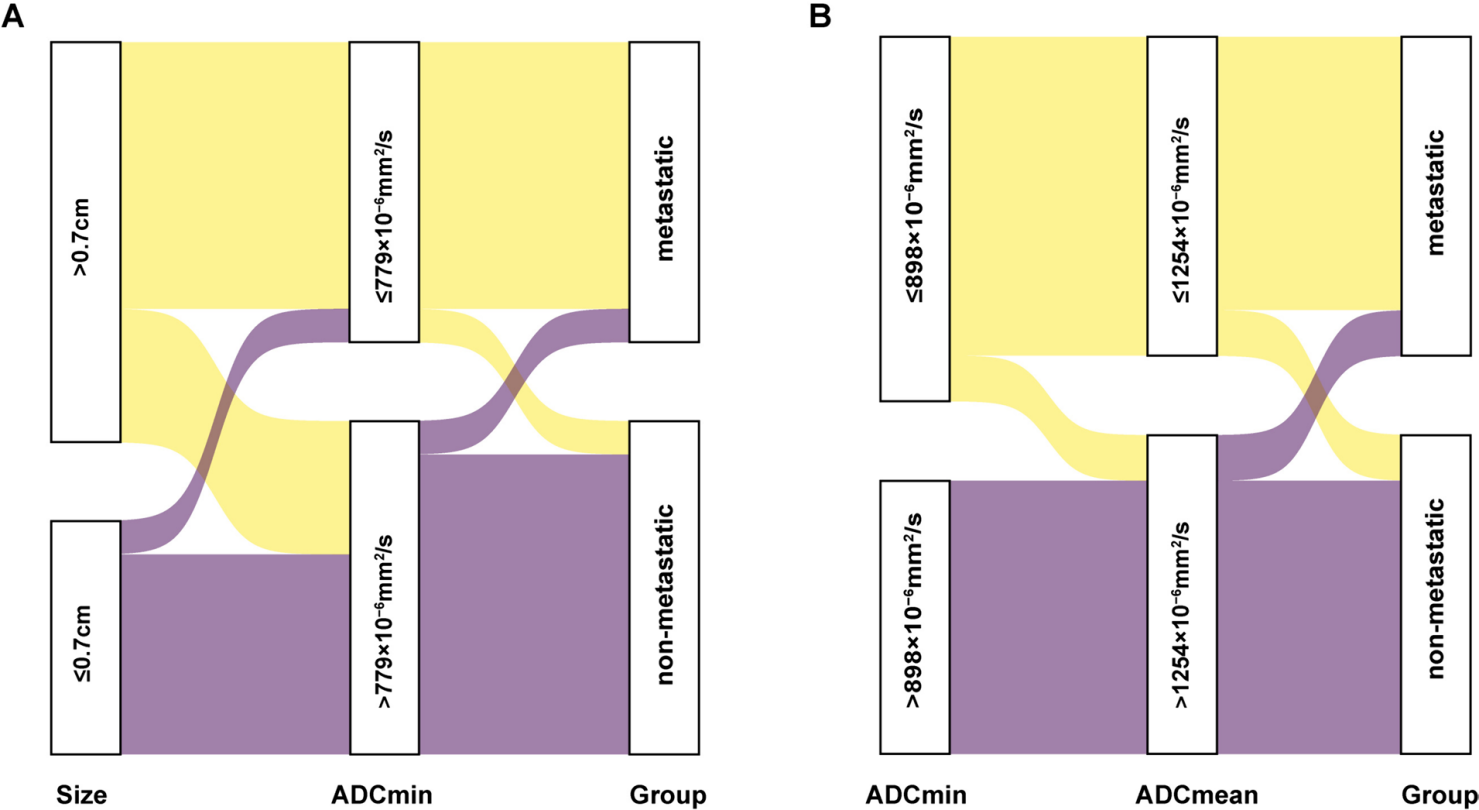
Sankey plot showing the relationships of ^68^Ga-DOTATATE PET/MR features in SSTR-RADS-3A and 3B. **A** LN size and ADCmin may improve SSTR-RADS-3A interpretation, while **B** ADCmin and ADCmean are more likely associated with SSTR-RADS-3B interpretation

Of the 14 SSTR-RADS-3B bone lesions evaluated in this study, follow-up results ultimately led to the classification of 7 as being malignant and 7 as being benign. Of the 7 metastatic lesions, 2 were osteolytic while 5 were osteogenic. With respect to the analyzed ADC parameters, metastatic lesions presented with significantly lower ADCmin values relative to non-metastatic lesions (618 ± 204 × 10^−6^mm^2^/s vs. 1077 ± 325 × 10^−6^mm^2^/s, P = 0.004). The ADCmean of metastatic lesions was also significantly lower than that for non-metastatic lesions (954 ± 340 × 10^−6^mm^2^/s vs. 1442 ± 396 × 10^−6^mm^2^/s, P = 0.03) (Supplemental Fig. 3). ROC analyses indicated that the optimal ADCmin cut-off threshold when differentiating between metastatic and non-metastatic bone lesions was 898 × 10^−6^mm^2^/s (P < 0.0001), yielding an AUC of 0.94 and respective sensitivity and specificity of 100% and 85.7%, respectively. ROC curves also suggested that the optimal ADCmean cut-off threshold for differentiating between metastatic and non-metastatic bone lesions was 1254 × 10^−6^mm^2^/s (P = 0.0015), with respective AUC, sensitivity, and specificity values of 0.86, 85.7%, and 85.7%, respectively (Fig. 4). The correlations of these predictive variables with SSTR-RADS 3A and 3B status as determined by ^68^Ga-DOTATATE PET/MR scans are shown in the Sankey plot (Fig. 5).

## Discussion

SSTR-PET is essential for diagnosing and treating NET patients, and vital for selecting peptide receptor radionuclide therapy (PRRT) candidates (Ambrosini et al. 2021). Increasing use of SSTR-PET for PRRT has prompted the creation of SSTR-RADS 1.0, a standard that enhances NET evaluation by integrating all imaging modalities (PET, CT, and hybrid imaging) to assess the total tumor burden more effectively (Werner et al. 2018). The SSTR-RADS classifications improve communication between radiologists and clinicians, help in patient risk stratification, and guide management decisions (Hope et al. 2018). In the present study, approximately 48.5% of uncertain lesions detected via ^68^Ga-DOTATATE PET/MR imaging were ultimately found to exhibit radiological changes consistent with tumor involvement on follow-up. Specifically, 47.4% of SSTR-RADS 3A LN lesions and 50.0% of SSTR-RADS 3B bone lesions met our established criteria for NET diagnosis. We selected SUVmax as the only semiquantitative parameter for evaluating LN and bone lesions due to its reproducibility and simplicity (Lee and Kim 2019). Unlike SUVmean, which varies with the VOI definition, SUVmax is less dependent on the observer. It is also less affected by partial volume effects (Soret et al. 2007). Moreover, precise SUVpeak measurements require a lesion diameter of at least 2 cm (Wahl et al. 2009), making it an unsuitable parameter for the small lesions in this study.

Metastatic LNs typically show active ^68^Ga-DOTATATE metabolism, LN enlargement, and elevated SUV (Ahmadi Bidakhvidi et al. 2021). In this study, LNs classified as SSTR-RADS 3A, whether metastatic or non-metastatic, had similar SUVmax values, likely due to their small size and the limited resolution of PET imaging (Xie et al. 2021). This highlights the importance of considering morphological changes. An LN short-axis diameter of 10 mm on CT and MR images is generally used to distinguish between benign and malignant nodes (Ganeshalingam and Koh 2009). In this cohort, metastatic LNs had larger diameters than non-metastatic ones, with a diameter cut-off of 0.7 cm providing 100.0% sensitivity and 60.0% specificity. Additionally, ADCmin values were significantly lower in metastatic LNs compared to non-metastatic LNs (584 ± 110 × 10^−6^mm^2^/s and 974 ± 192 × 10^−6^mm^2^/s, respectively; P < 0.001), assisting in differentiating their nature. Consistent with the findings of multiple studies on a variety of tumors, ADCmin was found to be effective for differentiating metastatic from non-metastatic LNs (Fardanesh et al. 2022; Liu et al. 2011; Li et al. 2015). Thus, ADCmin shows promise as an alternative parameter for the N-staging of NET patients exhibiting equivocal ^68^Ga-DOTATATE PET uptake.

The most common sites of NET bone metastasis include the bone marrow in the trunk, including the ribs, spine, and pelvis (Xie et al. 2022). In this study, we found such metastases primarily in the ribs and spine. Bone metastases can lead to severe symptoms including hypercalcemia, spinal cord compression, bone pain, and pathological fractures, which significantly worsen the patient's quality of life and prognosis (Kos-Kudła et al. 2010). Therefore, prompt and accurate diagnosis of bone metastases in NET patients is crucial to ensure effective treatment that can reduce pain and improve quality of life. Conventional imaging often falls short in accurately evaluating bone metastases. Hybrid imaging, which includes techniques like PET/CT and PET/MRI, overcomes these limitations by assessing morphological, functional, metabolic, and molecular characteristics in a single examination (Schmidkonz et al. 2019). In PET/CT imaging, the CT component is crucial for the evaluation of bone morphology and the identification of osseous destruction. MRI, used in PET/MRI hybrid imaging, offers superior soft-tissue contrast. The DWI sequence has proven effective for distinguishing bone metastases from both solid tumors and hematological malignancies (Pasoglou et al. 2015; Dimopoulos et al. 2015; Hottat et al. 2024). DWI also allows for quantifying lesion cellularity through ADC maps and numerous studies have demonstrated its value in bone metastases (Costelloe et al. 2021; Eveslage et al. 2023; Perez-Lopez et al. 2018). In our study, the ADCmin value for metastatic bone lesions was significantly lower than that for non-metastatic lesions (618 ± 204 × 10^−6^mm^2^/s vs. 1077 ± 325 × 10^−6^mm^2^/s, P = 0.004). The ADCmean of metastatic lesions was also lower than that of non-metastatic lesions (954 ± 340 × 10^−6^mm^2^/s vs. 1442 ± 396 × 10^−6^mm^2^/s, P = 0.03). In addition, ROC analyses revealed that the ADCmin and ADCmean parameters offered satisfactory diagnostic efficacy when classifying bone lesions, with respective AUC values of 0.94 and 0.86 such that they can be used to complement SSTR-RADS-3B classifications.

This study utilizes PET/MR imaging, which is not commonly available. Although PET/CT scanners are more widespread, there are instances where the SSTR-RADS 3A and 3B classifications may not conclusively determine the nature of a lesion. In such cases, additional MRI can be used to extract the ADC values of the lesion for predicting the outcome. If a hybrid PET/MR is unavailable, co-registered MR and PET-fused images can be used. Care is needed to avoid misregistration, particularly from patient movement. Misregistration is more likely with small LNs compared to truncal bones like the spine and pelvis. However, as long as the lesions are accurately aligned, the ADC values remain dependable.

This study has several limitations. Firstly, it was a retrospective analysis, which may introduce some degree of bias, and the number of indeterminate lesions was small, necessitating further validation of the cut-off values with a larger cohort. Secondly, the biopsy of indeterminate lesions detected via SSTR-targeted PET imaging is challenging due to their small size and difficulty in targeting with conventional imaging techniques, making histopathology infeasible. Follow-up imaging might be a more practical method for assessing these lesions. Thirdly, the study employed baseline PET/MR imaging with follow-up CT scans to evaluate SSTR-RADS-3B lesions but lacked baseline whole-body CT scans to provide information on detailed bone morphology.

## Conclusion

Of the SSTR-RADS-3A and SSTR-RADS-3B lesions exhibiting indeterminate SSTR-targeted PET uptake on initial imaging in this study, roughly half presented with changes consistent with NET involvement on follow-up imaging. Those SSTR-RADS-3A lesions exhibiting larger diameters and lower ADCmin values are more likely to correspond to metastases, while ADCmin and ADCmean can be leveraged to predict whether indeterminant bone lesions are metastatic or non-metastatic.

### Supplementary Information

Below is the link to the electronic supplementary material.

Supplementary file1 (DOCX 247 KB)

## Data Availability

All data in our study are available from the corresponding authors upon reasonable request.
