# Supplementary material for: Outcome prediction of SSTR-RADS-3A and SSTR-RADS-3B lesions in patients with neuroendocrine tumors based on 68Ga-DOTATATE PET/MR
Source: J Cancer Res Clin Oncol. 2024 May 25;150(5):272. doi: 10.1007/s00432-024-05776-5 (PMC11127844; doi:10.1007/s00432-024-05776-5)
Supplement: Supplementary file 1 — Supplementary file1 (DOCX 247 KB) [file 432_2024_5776_MOESM1_ESM.docx]

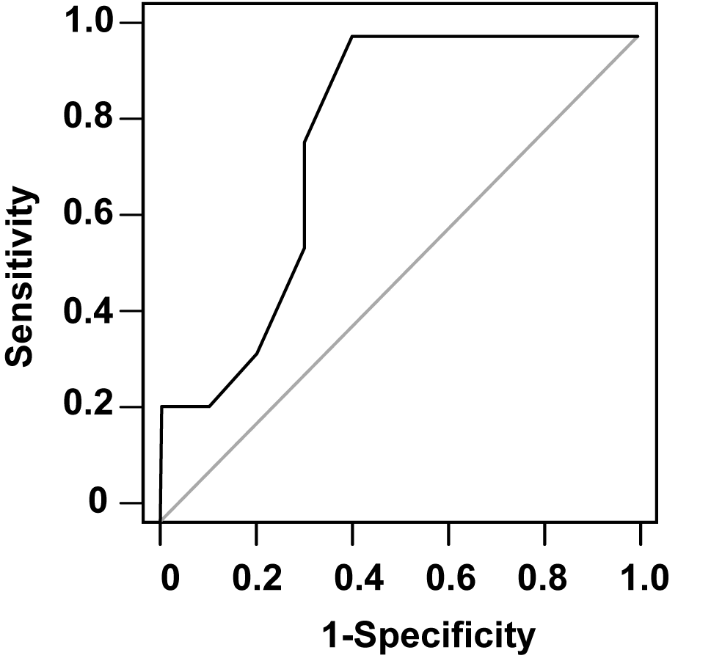


**Supplemental Fig.1**. ROC curve corresponding to evaluating LNs being benign or malignant based on LN size. LN: lymph node.

**
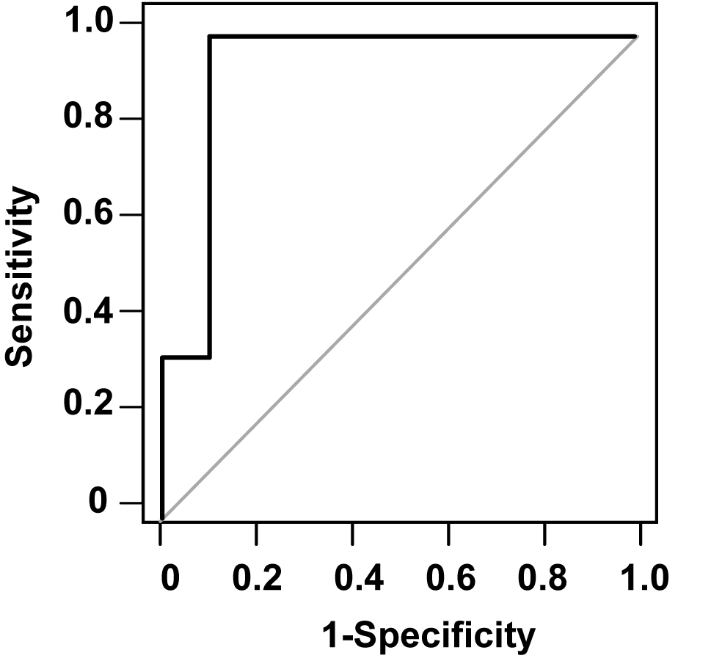
**

**Supplemental Fig.2**. ROC curve corresponding to the ADCmin for use differentiating between metastatic and non-metastatic LNs. The AUC value was 0.93.


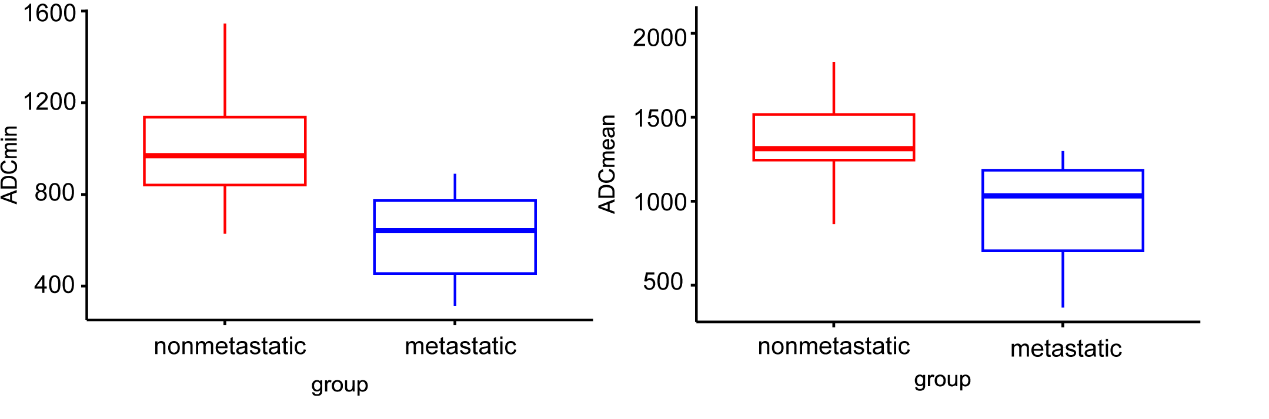


**Supplemental Fig.3**. Boxplots corresponding to the (A) ADCmin and (B) ADCmean values for metastatic and non-metastatic bone lesions.
